# Micellar Organocatalysis Using Smart Polymer Supports: Influence of Thermoresponsive Self-Assembly on Catalytic Activity

**DOI:** 10.3390/polym12102265

**Published:** 2020-10-01

**Authors:** Xiaoqian Yu, Artjom Herberg, Dirk Kuckling

**Affiliations:** Department of Chemistry, Paderborn University, Warburger Str. 100, D-33098 Paderborn, Germany; yu.xiaoqian1987@hotmail.com (X.Y.); artjom.herberg@uni-paderborn.de (A.H.)

**Keywords:** thermoresponsive block copolymers, azlactone ring-opening, immobilized L-prolineamide, RAFT polymerization, temperature-induced self-assembly, micellar organocatalysis

## Abstract

Micellar catalysts with a switchable core are attractive materials in organic synthesis. However, little is known about the role of the shell forming block on the performance of the catalyst. Thermoresponsive block copolymers based on poly(*N*-isopropylacrylamide-*co*-vinyl-4,4-dimethylazlactone) attached to different permanently hydrophilic blocks, namely poly(ethylene glycol), poly(*N*,*N*-dimethylacrylamide), and poly(2,3-dihydroxypropyl acrylate), were successfully synthesized via reversible addition/fragmentation chain transfer radical polymerization (RAFT). Post-polymerization attachment of an amino-functionalized L-prolineamide using the azlactone ring-opening reaction afforded functionalized thermoresponsive block copolymers. Temperature-induced aggregation of the functionalized block copolymers was studied using dynamic light scattering. It was shown that the chemical structure of the permanently hydrophilic block significantly affected the size of the polymer self-assemblies. The functionalized block copolymers were subjected to an aldol reaction between *p*-nitrobenzaldehyde and cyclohexanone in water. Upon temperature-induced aggregation, an increase in conversion was observed. The enantioselectivity of the polymer-bound organocatalyst improved with an increasing hydrophilic/hydrophobic interface as a result of the different stability of the polymer aggregates.

## 1. Introduction

Increasing sustainability in chemical reactions, chemical processes, and materials is one of the major challenges in modern times. The design of energy-efficient chemical reactions using recyclable catalysts and avoiding toxic reaction media are key aspects of green chemistry [1]. Catalyst immobilization mostly expands catalyst usability due to increased stability, improved solubility, and facilitated recycling [2,3,4,5]. Additionally, the use of stimuli-responsive polymers as catalyst supports offers the opportunity of reversibly tuning the catalyst activity by altering environmental parameters (temperature, pH, ionic strength, etc.) [6,7].

**Figure 1:**
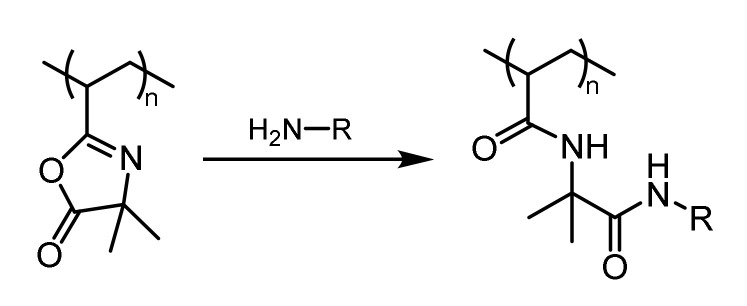
Ring-opening reaction of a primary amine with the azlactone group.

One strategy for catalyst immobilization involves the attachment of the catalyst to the polymeric carrier. Such post-polymerization modification of a polymer usually requires the presence of reactive functional groups. Functional polymers based on isocyanides [8], epoxides [9], active ester [10,11], anhydrides [12], and azlactones [13] were reported and utilized for different applications. Azlactone-functionalized polymers were of special interest because the azlactone moiety can be subjected to a rapid and atom-economic ring-opening reaction with different nucleophiles under mild reaction conditions. Exemplarily, the reaction of an amine with the azlactone moiety is depicted in Figure 1. Compared to active esters and anhydrides, the azlactone offers a better stability against hydrolysis, so the ring-opening reaction with good nucleophiles can be conducted in aqueous or alcoholic media, as well [14].

Incorporation of azlactone moieties into polymers is usually done by copolymerization with vinyl-4,4-dimethylazlactone (VDMA). The first synthesis of azlactone-functionalized polymers involved radical polymerization [15]. The development of reversible deactivation radical polymerization (RDRP) techniques offered the opportunity of synthesizing functional polymers of well-defined structure [16,17,18,19,20,21,22,23,24]. Narrow molar mass distribution, control over number-average molar mass, and control over polymer end groups are key features of RDRP and prerequisites for the synthesis of functional polymers with different architectures. Nitroxide-mediated radical polymerization (NMRP) [25], atom transfer radical polymerization (ATRP) [26,27,28,29], and reversible addition/fragmentation chain transfer radical polymerization (RAFT) [30] are commonly used RDRP techniques. The synthesis of well-defined poly(vinyl-4,4-dimethylazlactone) (PVDMA) by NMRP was first reported by Tully et al. [31]. Later, Fournier et al. used ATRP for the block copolymerization of VDMA with methyl methacrylate and styrene [32]. RAFT polymerization of VDMA yielding pH- and temperature-sensitive block copolymers was highlighted by Schilli et al. [33]. RAFT polymerization and temperature-induced self-assembly of thermoresponsive azlactone-containing block copolymers based on poly(*N*-isopropylacrylamide) (PNIPAAm) and poly(*N*,*N*-dimethylacrylamide) (PDMAAm) were described by Levere et al. [34]. Subsequent core-cross-linking via the azlactone groups using a diamine afforded stable nanoparticles. The use of a trithiocarbonate-modified poly(ethylene glycol) (PEG) as a macro-chain transfer agent for the RAFT copolymerization with *N*-isopropylacrylamide (NIPAAm) and VDMA was reported by Ho et al. in 2013 [35]. The azlactone moieties were also used for bioconjugation with lysozyme.

Catalysts based on low-molecular-weight, metal-free organic compounds have been known for more than a century. Studies on reaction mechanisms involving these so-called organocatalysts were presented by Pedersen and Westheimer in the 1930s and 1940s [36,37]. The first example of an asymmetric reaction using organocatalysts was reported by Hajos and Parrish in the 1970s [38]. Still, it took more than 30 years until the potential of this new class of asymmetric catalysts was recognized [39,40]. In 2000, List et al. reported the first proline-catalyzed direct asymmetric aldol reaction. Nowadays, proline and its derivatives represent the most widely used class of organocatalysts for enamine-type reactions [41,42,43]. Although L-proline can be considered a cheap compound, its use as an organocatalyst often requires catalyst loadings of more than 10 mol%. Additionally, the separation of L-proline after the reaction is often quite challenging. Substitution of L-proline by more active derivatives (e.g., prolineamides) significantly reduces the required catalyst loading. However, these derivatives are usually obtained by multiple-step synthesis. Thus, concepts of facilitated catalyst separation and recycling have gained increased attention [44]. In 1985, Kondo et al. reported the first type of polymer-supported proline. In the past decades, there have been numerous examples for the immobilization of proline on polymeric carriers like linear and cross-linked polymers, dendrimers, and polymeric capsules [45,46,47,48]. The use of stimuli-responsive polymer supports for proline immobilization was introduced in order to tune proline activity by altering ambient parameters [49,50]. For instance, the synthesis of a thermoresponsive block copolymer containing immobilized L-proline and its use in micellar catalysis were reported by O’Reilly and coworkers in 2013 [51]. Nevertheless, in most cases, the immobilization of proline on soluble polymer supports is usually done by copolymerization involving the synthesis of polymerizable proline derivatives, as described by Kristensen et al. [52]. Further, less is known about the role of the shell forming block on the performance of the catalyst.

**Figure 2:**
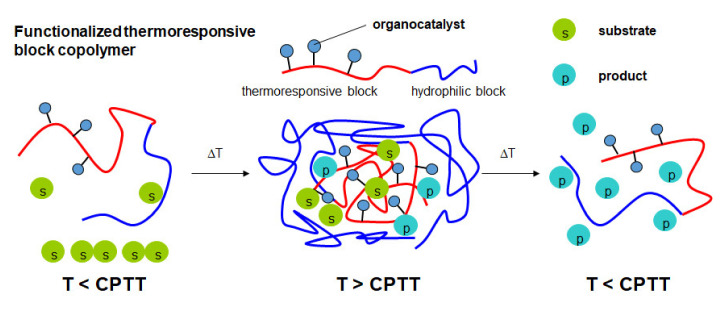
Basic principle of micellar organocatalysis using functionalized thermoresponsive block copolymers (CPTT = critical volume phase transition temperature).

Recently, our group reported the synthesis of amino-functionalized L-proline and different L-prolineamides. Subsequent immobilization of these amino-functionalized organocatalysts on azlactone-containing thermoresponsive block copolymers based on PEG and PNIPAAm afforded functional block copolymers for use in micellar organocatalysis [53]. In this strategy, the same polymer carrier was used for the immobilization of the different organocatalysts, thus allowing the comparison of catalyst activity and selectivity. Moreover, separation and recycling of an immobilized L-prolineamide organocatalyst were shown for up to five cycles. In the present work, this strategy is extended by synthesizing thermoresponsive azlactone-containing block copolymers with different permanently hydrophilic blocks, namely PEG, PDMAAm, and poly(2,3-dihydroxypropylacrylate) (PDHPA). Post-polymerization attachment of the prolineamide-based organocatalyst using azlactone ring-opening reaction affords the functionalized thermoresponsive block copolymers. By exceeding the critical volume phase transition temperature (CPTT), temperature-induced self-assembly of these block copolymers in aqueous solution creates nanoreactors capable of solubilizing the hydrophobic substrates (Figure 2). Spatial proximity between catalyst and substrate enhances the reaction rate. The effect of the different hydrophilic blocks on the catalyst activity and catalyst selectivity is investigated. The reaction can be stopped by decreasing the temperature below the CPTT, thus disassembling the nanoreactors and releasing the product.

## 2. Materials and Methods 

### 2.1. Characterization

Nuclear magnetic resonance (NMR) spectra (^1^H, ^13^C) were recorded on a Bruker AVANCE 500 (Bruker Scientific LLC, Billerica, MA, USA) instrument in deuterated solvents at 500 and 125 MHz, respectively. The chemical shifts δ in ppm are referenced to the respective solvent residual signal of CHCl_3_ (7.26 ppm) and DMSO (2.50 ppm). As solvents, chloroform-d_3_ (CDCl_3_, 99.8 D%) and dimethylsulfoxide-d_6_ (DMSO-d_6_, 99.5 D%) were used for NMR measurements. Data were processed using MestReNova Software (Mestrelab Research Chemistry Software Solutions, Santiago de Compostela, Spain). Size-exclusion chromatography (SEC) measurements were conducted on a modular SEC system running on tetrahydrofuran (THF) as an eluent. Two PSS-SDV columns (Polymer Standards Service (PSS), Mainz, Germany, 10^5^ and 10^3^ Å porosity with 5 μm particle size) were applied to separate the polymers at room temperature. Fractions were detected using a Knauer Smartline 2300 (Knauer, Berlin, Germany) refractive index detector. The flow rate was set to 1.0 mL/min and narrowly distributed poly(methyl methacrylate) (PMMA) standards were used for calibration. Data acquisition was accomplished using a PSS Universal Data center UDC 810 (Polymer Standards Service (PSS), Mainz, Germany). PSS WinGPC Unity software (Polymer Standards Service (PSS), Mainz, Germany) was used for data evaluation. High performance liquid chromatography (HPLC) measurements were performed on a Merck Hitachi D-Line 7000 (VWR International, Darmstadt, Germany) system equipped with a diode array ultraviolet detector at 254 nm. Sample separation was achieved using Chiralpak AD-H columns (from J. T. Baker B. V., Phillipsburg, NJ, USA) at 25 °C. The system was operated in an isocratic mode using 2-propanol and *n*-hexane (v/v 20/80) as an eluent at a flow rate of 0.8 mL/min. Freeze-drying of the polymer samples was performed on an Alpha 2-4 LDplus lyophilizer setup (Martin Christ, Osterode am Harz, Germany) at a −85 °C condenser temperature and 0.05 mbar pressure.

### 2.2. Materials

*N*-Isopropylacrylamide (NIPAAm, TCI Europe, Zwijndrecht, Belgium, >98%) was recrystallized from *n*-hexane and stored at −15 °C. *N*,*N*-Dimethylacrylamide (DMAAm, Sigma-Aldrich, St. Louis, MO, USA, 99%) was distilled under reduced pressure and stored at −15 °C. Azobis(isobutyronitrile) (AIBN, 98%) was obtained from Fluka (Fluka, Buchs, Switzerland) and recrystallized from methanol. Triethylamine (TEA, 99%) and cyclohexanone (CH, 99.8%) were purchased from Acros Organics (Acros Organics, Fair Lawn, NJ, USA) and used as received. *p*-Nitrobenzaldehyde (*p*-NBA, >98%) was obtained from Merck (Merck, Darmstadt, Germany) and used as received. The solvents methanol (99.5%), THF (99.5%) and 1,4-dioxane (99.5%) were obtained from Grüssing (Grüssing, Filsum, Germany) and were of analytical grade. 1,4-Dioxane was additionally stored over a 4 Å molecular sieve. Solvents for polymer precipitation (diethyl ether, *n*-hexane) were of technical grade.

The chain transfer agent 2-(dodecylthiocarbonothioylthio)-2-methylpropionic acid (DMP) was obtained according to a procedure by Lai et al. [54]. Modification of the PEG monomethyl ether (*M*_n_ = 5000 g/mol) with 4-cyano-4-pentanoate dodecyltrithio-carbonate was carried out as previously described [53]. Solketal acrylate (SKA) was synthesized by esterification of isopropylidene glycerol with acryloyl chloride according to literature [55,56]. *(S)*-*N*-(2-Aminoethyl) pyrrolidine-2-carboxamide dihydrochloride (L-prolinamide) was synthesized as previously described [53]. 2-Vinyl-4,4-dimethylazlacton (VDMA) was prepared via a two-step-synthesis described by Levere et al. [34].

### 2.3. Synthesis of the Block Copolymer PSKA-b-P(NIPAAm-co-VDMA)

A Schlenk tube with a septum and magnetic stirrer was charged with SKA (2.997 g, 16.1 mmol), DMP (117 mg, 0.322 mmol), AIBN (5 mg, 0.0322 mmol), and solvent 1,4-dioxane (5.3 mL) in the ratio of [SKA]_0_/[DMP]_0_/[AIBN]_0_ = 50/1/0.1. The resulting reaction mixture was purged with nitrogen for 20 min, before the Schlenk tube was immersed into an oil bath at 70 °C. After 24 h, the polymerization was stopped by freezing the reaction mixture with liquid nitrogen. After thawing, 1,4-dioxane was removed under reduced pressure and the polymer was isolated by precipitation in *n*-hexane at room temperature. The poly(solketal acrylate) (PSKA) was obtained as a sticky oil and used for the copolymerization as a macro-chain transfer agent.

For the copolymerization, the previously obtained PSKA-based macro-chain transfer agent (0.35 g), NIPAAm (1.5 g, 13.2 mmol), VDMA (0.15 g, 1.07 mmol), and AIBN (1 mg, 0.0121 mmol) were dissolved in 1,4-dioxane (5 mL). The reaction mixture was purged with nitrogen for 20 min, and the Schlenk tube was subsequently immersed into the oil bath. The polymerization proceeded at 70 °C for 24 h. Afterwards, the reaction was stopped by freezing the reaction mixture with liquid nitrogen. 1,4-Dioxane was removed under reduced pressure and the polymer was isolated by a series of precipitations in cold diethyl ether. The block copolymer was obtained as light-yellow powder after drying in vacuo.

^1^H-NMR (CDCl_3_, ppm): δ = 1.13 (br, 6H_PSKA_); 1.39 (br, 6H_PNIPAAm_); 1.57–2.49 (br, 3H_PSKA_, 3H_PNIPAAm_, 9H_PVDMA_); 3.88 (br, 1H_PNIPAAm_); 3.88–4.18 (br, 3H_PSKA_); 4.23–4.34 (br, 1H_PSKA_)

### 2.4. Synthesis of the Block Copolymer PDMAAm-b-P(NIPAAm-co-VDMA)

DMAAm (2.5 mL, 24.3 mmol), DMP (178 mg, 0.487 mmol), and AIBN (4 mg, 0.0487 mmol) were added to a Schlenk tube with a septum and magnetic stirrer, in the ratio of [DMAAm]_0_/[DMP]_0_/[AIBN]_0_ equal to 50/1/0.1. The resulting reaction mixture was purged with nitrogen for 20 min. Subsequently, the Schlenk tube was immersed into an oil bath. The polymerization proceeded overnight at 70 °C. The reaction was stopped by freezing of the mixture with liquid nitrogen. After thawing, the yellow solid reaction mixture was dissolved in THF (2 mL) and purified by precipitations in cold diethyl ether. After drying under fine vacuum, PDMAAm was obtained as a light-yellow powder.

For the copolymerization, the PDMAAm-based macro-chain transfer agent (0.5 g), NIPAAm (3.529 g, 31.19 mmol), VDMA (0.228 g, 1.64 mmol), and AIBN (1 mg, 0.0121 mmol) were dissolved in 1,4-dioxane (5 mL), and the resulting mixture was purged with nitrogen for 20 min. Polymerization was initiated by placing the Schlenk tube in the oil bath. The reaction proceeded overnight at 70 °C. The polymerization was stopped by freezing the reaction mixture with liquid nitrogen. 1,4-Dioxane was removed under reduced pressure and the polymer was purified by a series of precipitations in cold diethyl ether and *n*-hexane. The block copolymer was obtained as yellow powder after drying under fine vacuum.

^1^H-NMR (CDCl_3_, ppm): δ = 1.14 (br, 6H_PNIPPAm_); 1.43–2.35 (br, 3H_PDMAAm_, 3H_PNIPAAm_, 9_HPVDMA_); 2.90 (br, 6_HPDMAAm_); 4.00 (br, 1_HPNIPAAm_) 

### 2.5. Synthesis of the Block Copolymer PEG-b-P(NIPAAm-co-VDMA)

PEG-*b*-P(NIPAAm-*co*-VDMA) was synthesized following a previously described procedure [53]. A Schlenk tube with a septum and magnetic stirrer was charged with PEG-based macro-chain transfer agent (0.25 g, 0.045 mmol), AIBN (1 mg, 0.0121 mmol), NIPAAm (2.14 g, 18.94 mmol), VDMA (138 mg, 1 mmol), and dioxane (5.3 mL) in the ratio of [NIPAAm]_0_/[VDMA]_0_/[PEG-CDPA]_0_ = 420/20/1. The reaction mixture was purged with nitrogen for 20 min and the Schlenk tube was subsequently immersed into an oil bath at 70 °C to start the copolymerization. After 24 h, the reaction was stopped by freezing the reaction mixture with liquid nitrogen. 1,4-Dioxane was removed under reduced pressure, and the polymer was purified by a series of precipitations in cold diethyl ether and finally dried under fine vacuum.

^1^H-NMR (CDCl_3_, ppm): δ = 0.98–1.23 (br, 6H_PNIPAAm_); 1.46–2.40 (br, 3H_PNIPAAm_, 9H_PVDMA_); 3.63 (br, 4H_PEG_); 4.0 (br, 1H_PNIPAAm_)

### 2.6. Post-Polymerization Attachment of the Organocatalysts

Modification of the azlactone-containing thermoresponsive block copolymers was carried out as previously described [53]. In a 25 mL round-bottom flask, 0.5 g VDMA-containing amphiphilic block polymer (5 mol% VDMA) and 2 eq. of the amino-functionalized organocatalyst were dissolved in methanol (10 mL). TEA (0.3 mL) was slowly added and the resulting reaction mixture was stirred continuously overnight at room temperature. After the reaction, the clear solution was diluted with deionized water (5 mL) and low-molecular residuals were removed by dialysis against water at room temperature (Spectra Por^®^ 6 Dialysis Membrane MWCO 1000, Spectrum Chemical, New Brunswick, NJ, USA). The modified polymers were obtained as white powder after freeze-drying.

### 2.7. Hydrolysis of the Solketal Moieties

Deprotection of the solketal group was done according to a literature-known procedure [57]. A mass of 0.5 g of the PSKA-*b*-P(NIPAAm-*co*-VDMA-prolinamide) block copolymer was dissolved in a mixture of 10 mL THF and 15 mL glacial acetic acid. The solution was heated to 90 °C and stirred for 6 h. During the reaction time, 24 mL deionized water was added portion-wise. The solvent was evaporated, residual acetic acid was removed by subsequent dialysis against 0.01 M sodium hydroxide solution and water (Spectra Por^®^ 6 dialysis membrane MWCO 1000, Spectrum Chemical, New Brunswick, NJ, USA), and the resulting polymer solution was freeze-dried.

### 2.8. Temperature-Induced Aggregation of the Block Copolymers

A mass of 5 mg of the amphiphilic block copolymer was dissolved in 10 mL of distilled water. Being left for stirring overnight at room temperature, the resulting solution was subsequently filtered through a syringe filter (0.45 μm, poly(tetrafluoroethylene, PTFE). Then, 3 mg of *p*-NBA and 12 µL CH were added, and the mixture was stirred for 10 min prior to being filled into a cuvette. Dynamic light scattering analysis afforded the particle size as a function of temperature. The measurements were performed on a Zetasizer ZS from Malvern Instruments (Malvern PANalytical, Malvern, UK). The instrument was operated in backscattering mode. The temperature program covered a range of 20 °C to 65 °C in intervals of 1 K. The measurements were carried out 3 times at each temperature.

### 2.9. Asymmetric Aldol Reaction in Water

The amount of functionalized block copolymers used for the aldol reaction depends on the relative block copolymer composition and the molar mass of the organocatalyst (Table 3). In general, 30 mg (200 µmol) *p*-NBA, 120 μL (1.16 mmol) CH, and a functionalized block copolymer carrying 16 µmol immobilized organocatalyst were suspended in deionized water. The resulting reaction mixture was stirred continuously for 10 min at room temperature and subsequently immersed into an oil bath at a certain temperature to start the catalytic reaction. After the reaction, the reaction mixture was diluted with water (10 mL) and extracted 3 times with diethyl ether. The organic phase was dried over MgSO_4_ and the solvent was removed under reduced pressure. The crude product mixture was analyzed using ^1^H-NMR spectroscopy and HPLC on a chiral stationary phase. The functionalized block copolymers dissolved in an aqueous phase can be isolated by freeze-drying.

## 3. Results and Discussion

### 3.1. Synthesis of the Functionalized Block Copolymers

**Figure 3:**
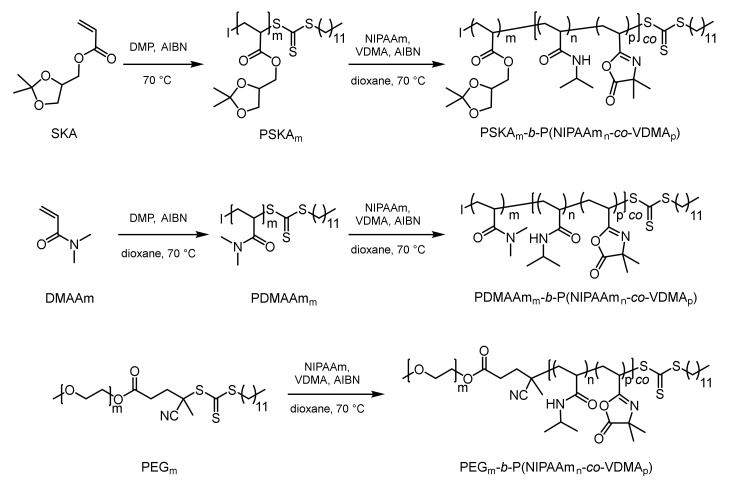
Synthesis of the azlactone-containing thermoresponsive block copolymers with different hydrophilic blocks (I = initiating group).

Three different block copolymers were synthesized by RAFT polymerization. For all three block copolymers, the thermoresponsive block comprised a copolymer of NIPAAm and VDMA. Meanwhile, the PNIPAAm provided thermoresponsivity, and the azlactone moiety was used for post-polymerization attachment of an amino-modified organocatalyst. The chemical nature of the hydrophilic block was varied from PDHPA and PDMAAm to PEG. Block copolymer synthesis started with the formation of the hydrophilic block (Figure 3). According to that, SKA and DMAAm were polymerized separately under RAFT conditions using the trithiocarbonate-based chain transfer agent DMP. Due to the controlled polymerization process, the resulting PSKA and PDMAAm exhibited an active trithiocarbonate end group at the ω-chain end. Thus, these homopolymers were used as macro-chain transfer agents in the subsequent copolymerization of NIPAAm and VDMA, yielding block copolymers of the desired structure. For the synthesis of the third copolymer, monomethoxy-PEG with a number-average molar mass of 5000 g/mol was modified with a carboxylic acid-containing trithiocarbonate, as previously described [53]. The degree of functionalization was found to be higher than 95%. The resulting PEG macro-chain transfer agent was also utilized in the copolymerization of NIPAAm and VDMA, affording the desired block copolymer. The azlactone content in the thermoresponsive block was targeted to be 5 mol% in order to achieve a reasonable amount of immobilized organocatalyst but still maintain thermoresponsive behavior with a CPTT near 32 °C.

The thermoresponsive azlactone-containing block copolymers were characterized using ^1^H-NMR spectroscopy and SEC (Table 1). The relative block length ratio of the hydrophilic and the thermoresponsive block varied from 1.0/3.1 and 1.0/6.8 to 1.0/8.7 according to the hydrophilic block. Discher and Eisenberg found out that the morphology of the self-assembled polymers in aqueous solution depends on the weight ratio between the hydrophilic and hydrophobic block [58]. Amphiphilic block copolymers with a mass fraction of either <25 w% or >45 w% according to the hydrophilic block are expected to form small micellar aggregates. In the present study, the mass fraction of the thermoresponsive block was chosen to be in the range of 85–90 w% to obtain a clear and fast volume phase transition. Additionally, Liu at el. reported that a higher mass fraction of the permanently hydrophilic block leads to a restricted mass transfer in the micellar catalysis, thus decreasing the reaction rate [59]. The number average molar masses (*M*_n_) of the block copolymers range between 39.7 and 55.9 kg/mol relative to a calibration based on PMMA standards. Although the molar mass dispersity seems to be high (*Ð* > 1.40), the molar mass distributions of the block copolymers are uniform (see Figure S1). Only the molar mass distribution of the PEG-based block copolymer showed a small shoulder attributed to the residual fraction of unreacted PEG chains as a consequence of incomplete functionalization with the chain transfer agent. The broadness of the molar mass distributions can be explained by the significant difference in reactivity of the NIPAAm and VDMA monomers. Furthermore, the DMAAm monomer is very reactive, leading to a broadly distributed PDMAAm macro-chain transfer agent.

**Figure 4:**
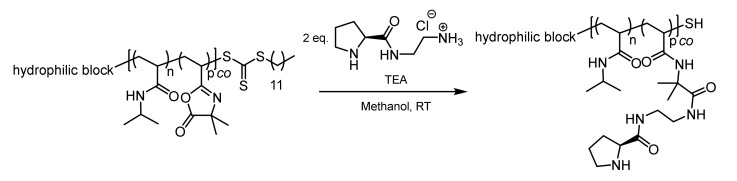
Post-polymerization attachment of an amino-functionalized L-prolinamide to the azlactone-containing thermoresponsive block copolymers.

The azlactone functionality is capable of undergoing a ring opening reaction in the presence of nucleophiles. The chemical reactivity of the azlactone group is determined by the presence of multiple electrophilic functional groups (C=O, C=C, and C=N). The slightly exothermic ring opening reaction will take place with primary amines and thiolates in the absence of any catalyst. In the present work, the post-polymerization modification of PVDMA was carried out using an amino-functionalized L-prolineamide (Figure 4). According to the synthetic route, final deprotection afforded the amino-functionalized organocatalysts as a hydrochloric acid adduct. To restore the nucleophilicity of the amino group, the hydrochloric acid was neutralized by stoichiometric amounts of TEA. The resulting triethylamine hydrochloride salt can be easily removed by dialysis against water. Methanol turned out to be the best solvent for the post-polymerization attachment of the organocatalyst because methanol is capable of dissolving both the polymer and the hydrochloric adduct of the L-prolinamide. In order to reduce the competitive reaction between PVDMA and methanol, the post-polymerization modification was carried out at room temperature in the presence of an excess of the organocatalyst hydrochloric adduct and a stoichiometric amount of TEA. Furthermore, ^1^H-NMR spectroscopy, as well as UV–VIS spectroscopy, revealed the loss of the trithiocarbonate functionality during the post-polymerization modification. The vanishing of the characteristic UV absorption of the trithiocarbonate is exemplarily shown for the sample POC 3 (see Figure S5). The reaction of thiocarbonylthio compounds with nucleophiles is one of the most widely reported methods to convert RAFT end groups. The free primary amine attacks the thiocarbonylthio groups in an amidation reaction, thereby releasing the thiol end group in the polymer. In this way, the hydrophobic dodecyl group, which might have an influence on the self-assembly process, could be removed.

**Figure 5:**
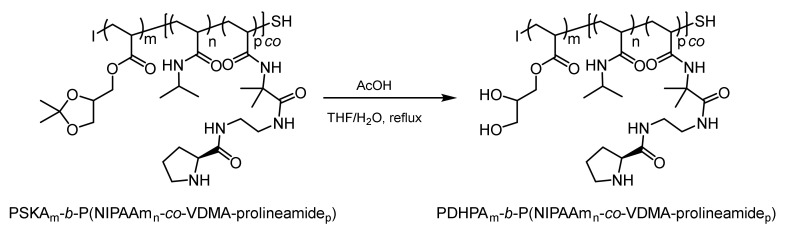
Deprotection of the solketal moiety affording the hydrophilic poly(2,3-dihydroxypropylacrylate) (PDHPA) block (AcOH = glacial acetic acid).

Finally, the solketal moiety was hydrolyzed using glacial acetic acid, thus yielding the hydrophilic PDHPA block (Figure 5). The success of this deprotection was proved by ^1^H-NMR spectroscopy, as previously reported [57].

At this step, three thermoresponsive block copolymers bearing the immobilized L-prolineamide organocatalyst and comprising three chemically different permanently hydrophilic blocks were available (Table 2). As the most important feature of the modified block copolymers, the catalyst loading of the copolymers was analyzed by ^1^H-NMR spectroscopy (Table 3, Figures S2–S4). The molar copolymer composition of PEG-*b*-P(NIPAAm-*co*-VDMA-prolinamide) was calculated by comparing the characteristic signals corresponding to C*H* of PNIPAAm (at 3.84 ppm), the C*H*_2_-C*H*_2_ of PEG (at 3.51 ppm), and the C*H*_2_ of the proline-moiety (at 2.75 ppm). The molar copolymer composition of PDHPA-*b*-P(NIPAAm-*co*-VDMA-prolinamide) was calculated by comparing the characteristic signals corresponding to C*H* of PNIPAAm (at 3.84 ppm), the O*H* of PDHPA (at 4.59 and 4.78 ppm), and the C*H*_2_ of the proline-moiety (at 2.87 ppm). For the modified block copolymer PDMAA-*b*-P(NIPAAm-*co*-VDMA-prolinamide), the molar composition was determined by comparing the integral under the peaks attributed to the C*H* of PNIPAAm (at 3.84 ppm), the C*H*_3_ of PDMAA (at 2.79 ppm), and the C*H* of proline-moiety (at 3.51 ppm).

The molar content of the immobilized L-prolinamide organocatalyst was found to be in the range of 3–5 mol%. However, considering a targeted azlactone content of 5 mol%, the experimentally verified organocatalyst content of 3–5 mol% indicates that the nucleophilic ring-opening reaction of the azlactone by the organocatalyst did not proceed quantitatively. Approximately 60–95% of the available azlactone moieties were functionalized with the organocatalyst. Still, this degree of functionalization seems reasonable taking into account that the reaction was conducted in methanol. The application of the immobilized organocatalysts to an asymmetric aldol reaction in water requires knowledge of the catalyst loading, which is the number ratio of catalyst to substrate. Due to the dispersity in molar mass, polymer concentrations are usually mass-related. Therefore, the mass fraction of the organocatalyst was also calculated based on the molar block copolymer composition. Thus, the mole number of immobilized organocatalyst can be calculated from the applied polymer mass. According to the differences in the organocatalyst structure, the mass-related catalyst content differs between 9 and 12% w/w.

### 3.2. Temperature-Induced Self-Assembly of the Functionalized Thermoresponsive Block Copolymers

Thermoresponsive polymers undergo significant changes in their properties due to small changes in ambient temperature [60]. Thereby, changes in molecular interactions lead to macroscopic changes of the polymer material, such as dimension, mechanical and optical properties, or permeability. PNIPAAm is the best investigated thermoresponsive polymer showing lower critical solution temperature (LCST) behavior [61]. It means that in aqueous solution, PNIPAAm can be shifted from a hydrophilic to a hydrophobic state by heating the solution beyond a certain temperature called the critical volume phase transition temperature (CPTT). This process is reversible; thus, upon cooling the solution below CPTT, PNIPAAm becomes water-soluble again. Block copolymers containing a hydrophilic block and a thermoresponsive PNIPAAm block can be reversibly turned from a totally hydrophilic into an amphiphilic macromolecule by exceeding the CPTT. Therefore, such thermoresponsive block copolymers belong to the double hydrophilic block copolymers [62]. The amphiphilic structure will lead to a temperature-induced self-assembly of the macromolecules. Molecular parameters like molar mass, block length ratio, and polymer architecture are known to affect the size, morphology, and colloidal stability of the self-assembled structures in solution [62]. Besides, the chemical structure of the permanently hydrophilic block should have significant influence on the stabilization of the polymer aggregates, as well. If these aggregates are used as nanoreactors by incorporating catalytically active moieties and loading with proper substrates, the stabilizing properties of the amphiphilic block copolymers will affect the proceeding of the chemical reaction inside the core of the polymer aggregates. According to this, the temperature-induced aggregation of the functionalized block copolymers in aqueous media containing the reactants *p*-nitrobenzaldehyde (*p*-NBA) and cyclohexanone (CH) was studied using dynamic light scattering (DLS). The overall ratio between functionalized block copolymer, *p*-NBA, and CH in this heterogeneous system matched the conditions for the later organocatalytic reactions. However, the overall concentrations had to be decreased in order to achieve evaluable scattering intensities in the DLS experiment.

**Figure 6:**
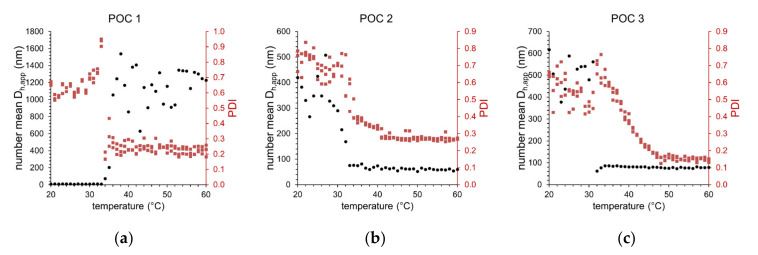
Development of the number mean apparent hydrodynamic diameter (*D*_h,app_) and polydispersity index (PDI) as a function of solution temperature for (**a**) POC 1, (**b**) POC 2, and (**c**) POC 3 thermoresponsive block copolymers carrying the immobilized organocatalyst in aqueous media containing the substrates *p*-NBA and CH (dot = number mean hydrodynamic diameter, square = PDI).

Figure 6 shows the results of the temperature-dependent DLS measurements for the three different block copolymers carrying the L-prolinamide-based organocatalyst. Obviously, there is a significant difference in the temperature-dependent behavior of the number mean hydrodynamic diameter (*D*_h,app_, black dots) between sample POC 1 on the one hand and samples POC 2 and POC 3 on the other hand. For POC 1, at low temperatures, mainly small species with *D*_h,app_ ≈ 10 nm can be observed. Around the CPTT of 35 °C, a rapid increase in *D*_h,app_ shows the formation of larger aggregates with 600–1400 nm in size. The extensive scattering of *D*_h,app_ indicates the presence of differently sized species. By contrast, for samples POC 2 and POC 3, a formation of larger aggregates 250–600 nm in size at temperatures below CPTT can be seen. Around the CPTT, a significant decrease in *D*_h,app_ indicates the formation of smaller particles with less than 100 nm in size. The CPTT for samples POC 2 and POC 3 is around 32 °C. The broadness of particle size distributions characterized by the polydispersity index (PDI, red dots) is significantly reduced by exceeding the CPTT for all three samples. Thus, above CPTT, particles with narrower size distributions are formed. The smallest PDI value of 0.15 was observed for sample POC 3 at temperatures above 50 °C. Table 4 summarizes the characteristic values for the temperature-induced aggregation of the functionalized block copolymers at two temperatures: 25 °C (below CPTT) and 40 °C (above CPTT). The observed deviation in aggregation behavior indicates that the three functionalized block copolymers exhibit different capability of stabilizing aqueous emulsions. The reactants *p*-NBA and CH are almost insoluble in water, thus leading to a heterogeneous system. However, the functionalized block copolymers POC 2 and POC 3 are capable of stabilizing larger substrate droplets even below CPTT. Therefore, an emulsion with larger droplets can be seen in the DLS experiment. Due to the amphiphilic character of the block copolymers above CPTT, the stabilization of the hydrophobic substrate domains becomes even better, resulting in the formation of micelles solubilizing the hydrophobic substrates. The PEG-based functionalized block copolymer POC 1 cannot stabilize the hydrophobic substrates below CPTT. Hence, mainly single dissolved block polymer chains with *D*_h,app_ ≈ 10 nm can be seen. Above the CPTT, the amphiphilic character improves the stabilizing properties of the block copolymer, leading to the formation of stabilized substrate domains. However, the dimension of these stabilized substrate domains is much larger compared to the micellar systems of POC 2 and POC 3.

### 3.3. Micellar Organocatalysis in Water

**Figure 7:**
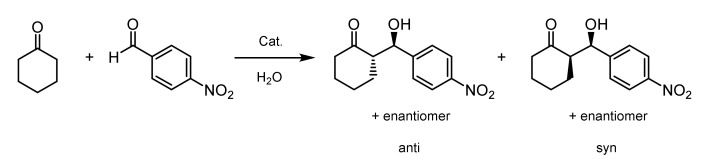
Aldol reaction between *p*-NBA and CH catalyzed by the thermoresponsive block copolymers carrying the L-prolineamide-based organocatalyst.

Finally, the functionalized block copolymers were subjected to an organocatalytic aldol reaction between *p*-NBA and CH at different temperatures (Figure 7). This reaction affords four stereoisomers: Two pairs of enantiomers and two pairs of diastereomers (anti/syn). The diastereomeric ratio (*dr*) was determined by ^1^H-NMR spectroscopy and HPLC. The enantiomeric excess (*ee*) of the anti and syn adducts was determined by HPLC using a chiral stationary phase. The reactions were carried out at 25 °C and 40 °C, which are below and above the CPTT of the functionalized block copolymers, respectively. The molar ratio of immobilized organocatalyst to *p*-NBA was chosen to be 8 mol%. Table 5 summarizes the results for the organocatalytic aldol reaction. It is obvious that below the CPTT, all the reactions proceeded much slower, leading to *p*-NBA conversions of 10% at maximum within 24 h. Above the CPTT, a significant increase in the *p*-NBA conversion was achieved. By reducing the volume of water and, thereby, increasing the concentration of substrates and functionalized block copolymers, the conversion could be optimized to 74% for POC 1 and 83% for POC 2 and POC 3. The diastereomeric ratio of the products did not show a significant dependency on temperature or concentration, whereas the *ee* values significantly improved by raising the temperature above the CPTT and increasing the concentration of the substrate and functionalized block copolymer. Gruttadauria et al. reviewed the role of water for organocatalytic reactions [63]. According to that, water plays a crucial role for the activity and stereoselectivity of proline-based catalysts. Highly stereoselective reactions could be achieved at the interphase between hydrophobic and aqueous domains. In particular, the micellar systems formed by the samples POC 2 and POC 3 above the CPTT exhibit a large interfacial area between the hydrophobic substrates and the aqueous phase. Due to its immobilization in the thermoresponsive block, the organocatalyst is located at the interface area, thereby showing a very high enantioselectivity. Previous studies revealed that even in the presence of the unattached organocatalyst, there is no observable aldehyde conversion after 24 h [53]. The lower *ee* values of POC 1 can be attributed to the larger aggregates formed above CPTT and the resulting low interface area.

## 4. Conclusions

Thermoresponsive block copolymers based on PNIPAAm attached to different permanently hydrophilic blocks, namely PEG, PDMAAm, and PDHPA, were successfully synthesized via RAFT polymerization. Additionally, the thermoresponsive block contained azlactone moieties used for post-polymerization attachment of an amino-functionalized organocatalyst based on L-prolinamide. These functionalized thermoresponsive block copolymers were subjected to the aldol reaction between *p*-NBA and CH in aqueous media. Dynamic light scattering revealed that temperature-induced self-assembly of the functionalized block copolymers lead to the formation of nanoreactors with the hydrophobic substrates in the core and the immobilized organocatalyst at the hydrophilic/hydrophobic interface. However, the dimension of the formed polymer aggregates significantly depended on the chemical structure of the permanently hydrophilic blocks. Whereas the presence of PEG as a stabilizing block afforded larger aggregates (*D*_h,app_ ≈ 800 nm), the use of PDMAAm and PDHPA as a hydrophilic block led to the formation of micellar aggregates (*D*_h,app_ < 100 nm). The temperature-induced self-assembly of the block copolymers significantly enhanced the reaction rates of the aldol reaction. Diastereoselectivity of the immobilized catalyst was not affected by polymer self-assembly, whereas enantioselectivity clearly improved upon the formation of the nanoreactors. The size of the polymer aggregates also affected the *ee* values. Within the micellar aggregates, higher *ee* values were observed compared to the larger aggregates. This effect can be attributed to the larger hydrophilic/hydrophobic interface formed in the micellar system, thus improving stereoselectivity of the organocatalyst.

## Figures and Tables

**Table 1:** Characterization of the azlactone-containing thermoresponsive block copolymers with different hydrophilic blocks.

Sample	*m*/*n*^a^ (NMR)	Weight Ratio *m*/*n*	*Đ* ^b^	*M*_n,_^b^ (g/mol)
PSKA_m_-*b*-P(NIPAAm_n_-*co*-VDMA_p_)	1.0: 8.7	1.0: 6.8	1.49	46,800
PDMAAm_m_-*b*-P(NIPAAm_n_-*co*-VDMA_p_)	1.0: 6.8	1.0: 7.8	1.91	55,900
PEG_m_-*b*-P(NIPAAm_n_-*co*-VDMA_p_)	1.0: 3.1	1.0: 8.1	1.43	39,700

^a^ Determined using ^1^H-NMR spectroscopy. ^b^ Determined by SEC in THF with PMMA standard calibration.

**Table 2:** Structure of the polymer-bound organocatalyst (POC) after post-polymerization attachment of the L-prolineamide.

Sample	Polymer Composition
POC 1	PEG_m_*-b-*P(NIPAAm_n_-*co*-VDMA-prolinamide_p_)
POC 2	PDHPA_m_*-b-*P(NIPAAm_n_-*co*-VDMA-prolinamide_p_)
POC 3	PDMAA_m_*-b-*P(NIPAAm_n_-*co*-VDMA-prolinamide_p_)

**Table 3:** Copolymer composition and catalyst loading of the amphiphilic block copolymers bearing the immobilized organocatalyst.

Sample	*m*/*n*/*p* (molar) ^a^	Prolineamide-Content ^b^ (mol%)	m*/n*/p* (mass) ^c^	Prolinamide-Content ^d/^ (% w/w)
POC 1	1.00/3.09/0.13	3	44/349/42	10
POC 2	1.00/10.00/0.53	5	140/1130/157	12
POC 3	1.00/6.40/0.26	3	99/712/80	9

^a^ Calculated from ^1^H-NMR spectroscopy hydrophilic/PNIPAAm/prolinamide-moiety molar ratio. ^b^ Prolinamide molar loading was calculated by *p*/(*m* + *n* + *p*) × 100%. ^c^ mass-related copolymer composition calculated by *m* × *M*_hydrophilic_/*n* × *M*_NIPAAm_/ *p* × *M*_VDMA-Prolinamide_. ^d^ mass-related prolinamide-content calculated by p*/(m*+n*+p*) × 100%.

**Table 4:** Characteristic parameters for the temperature-induced aggregation of the thermoresponsive block copolymers carrying the immobilized organocatalyst in aqueous media containing the substrates *p*-NBA and CH.

Sample	CPTT (°C)	25 °C		40 °C	
		PDI	Number Mean *D*_h,app_ (nm)	PDI	Number Mean *D*_h,app_ (nm)
POC 1 + substrates	35	0.65	11	0.25	856
POC 2 + substrates	32	0.67	425	0.34	74
POC 3 + substrates	32	0.51	588	0.38	82

**Table 5:** Data for the organocatalytic aldol reaction of CH and *p*-NBA after 24 h reaction time, using the thermoresponsive block copolymers carrying the L-prolineamide-based organocatalyst.

Catal.	*T* °C	*V*_(H2O)_ (mL)	n(Catalyst)/ n(*p*-NBA) (%)	Conv. (%)	*dr*^a,b^ (Anti/Syn) NMR; HPLC	*ee*^b^ (%) (Anti; Syn)
POC 1	25	3	8.1	10	75/25; 75/25	14; 23
	40	3	8.1	58	83/17; 77/23	20; 6
	40	1	8.1	74	77/23; 79/21	33; 1
POC 2	25	3	8.0	6	85/15; 80/20	43; 22
	40	3	8.0	55	80/20; 75/25	60; 23
	40	1	8.0	83	81/19; 65/35	82; 40
POC 3	25	3	8.0	10	85/15; 80/20	23; 25
	40	3	8.0	39	82/18; 77/23	45; 2
	40	1	8.0	83	80/20; 59/41	61; 64

^a^ Calculated from ^1^H-NMR spectroscopy; ^b^ determined by HPLC, AD-H column, isopropanol/*n*-hexane (1/9).

## Supplementary Materials

The following are available online at https://www.mdpi.com/2073-4360/12/10/2265/s1, Figure S1: Normalized molar mass distributions of the azlactone-containing block copolymers (SEC in THF, PMMA calibration); Figure S2: ^1^H-NMR spectrum of the functionalized block copolymer POC 1 (characteristic peaks are labelled, integrated and assigned); recorded at 500 MHz in DMSO-d6; Figure S3: ^1^H-NMR spectrum of the functionalized block copolymer POC 2 (characteristic peaks are labelled, integrated and assigned); recorded at 500 MHz in DMSO-d6; Figure S4: ^1^H-NMR spectrum of the functionalized block copolymer POC 3 (characteristic peaks are labelled, integrated and assigned); recorded at 500 MHz in DMSO-d6; Figure S5: UV/VIS spectra of the DMP RAFT agent and the PDMAAm-*b*-P(NIPAAm-*co*-VDMA) block copolymer before and after attachment of the prolineamide organoctatalyst (solvent: methanol)
