# Supplementary material for: Micellar Organocatalysis Using Smart Polymer Supports: Influence of Thermoresponsive Self-Assembly on Catalytic Activity
Source: Polymers (Basel). 2020 Oct 1;12(10):2265. doi: 10.3390/polym12102265 (PMC7600719; doi:10.3390/polym12102265)
Supplement: Supplementary file 1 [file polymers-12-02265-s001.pdf]

Micellar Organocatalysis Using Smart Polymer Supports: Influence Of Thermoresponsive Self-Assembly On Catalytic Activity

Supporting Information

*Xiaoqian Yu, Artjom Herberg, Dirk Kuckling**

Paderborn University, Department of Chemistry, Warburger Str. 100, D-33098 Paderborn, Germany

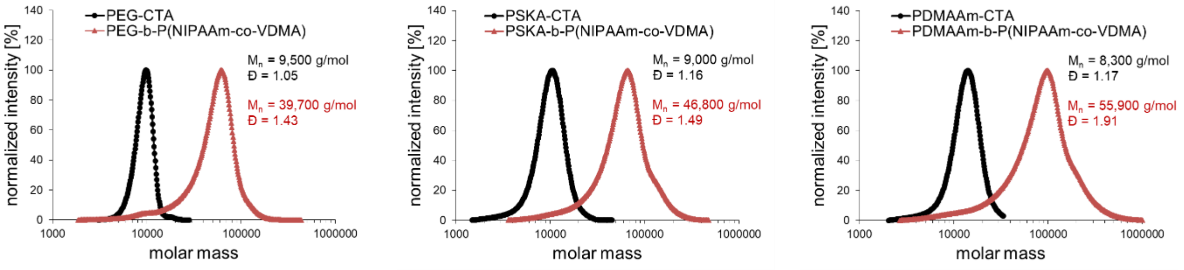

FIGURE S1: Normalized molar mass distributions of the macro-RAFT agent and the resulting azlactone-containing block copolymers (SEC in THF, PMMA calibration)

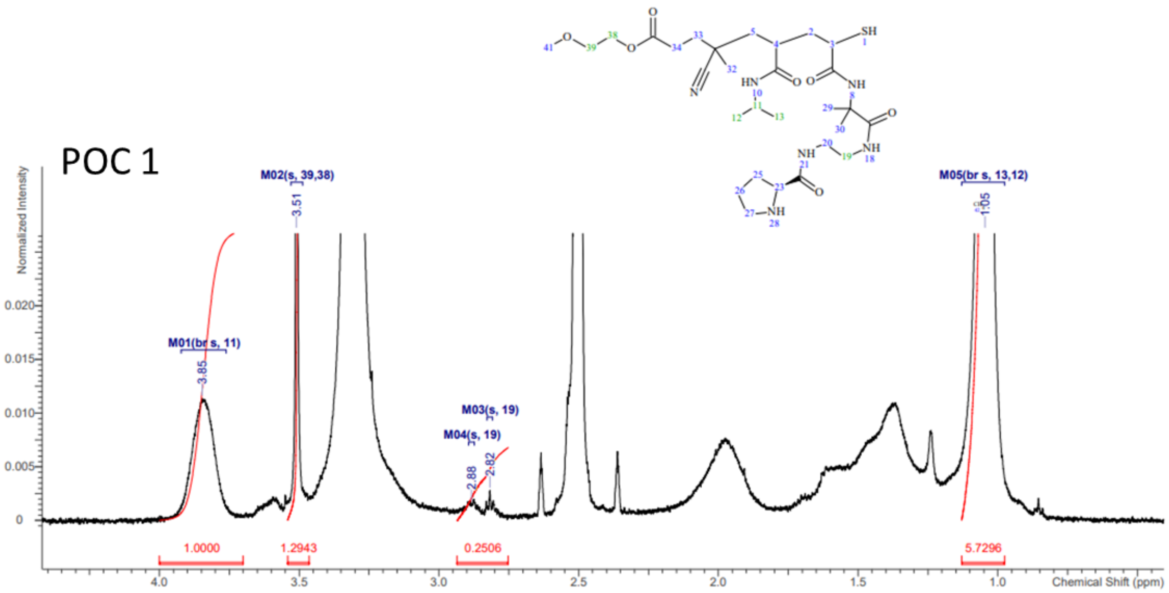

FIGURE S2: ^{1}H-NMR spectrum of the functionalized block copolymer POC 1 (characteristic peaks are labelled, integrated and assigned); recorded at 500 MHz in DMSO-d6

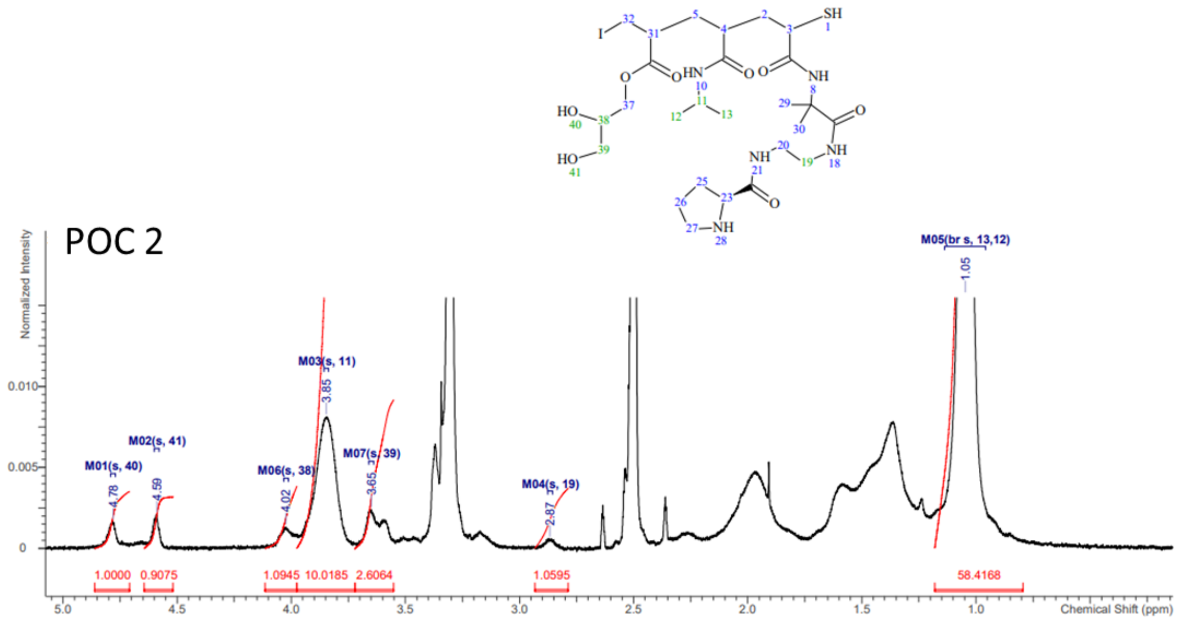

FIGURE S3: ^{1}H-NMR spectrum of the functionalized block copolymer POC 2 (characteristic peaks are labelled, integrated and assigned); recorded at 500 MHz in DMSO-d6

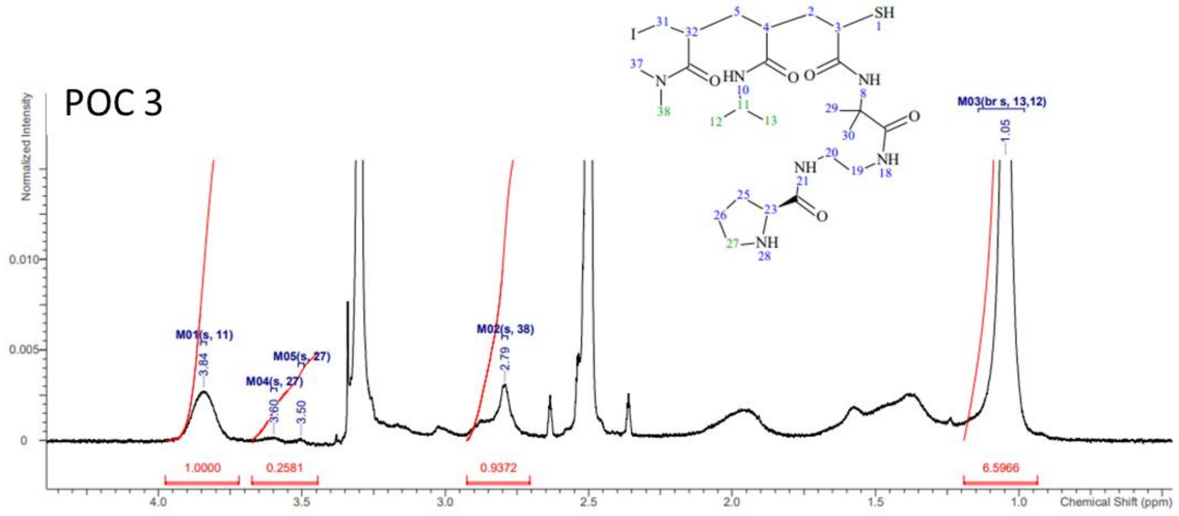

FIGURE S4: ^{1}H-NMR spectrum of the functionalized block copolymer POC 3 (characteristic peaks are labelled, integrated and assigned); recorded at 500 MHz in DMSO-d6

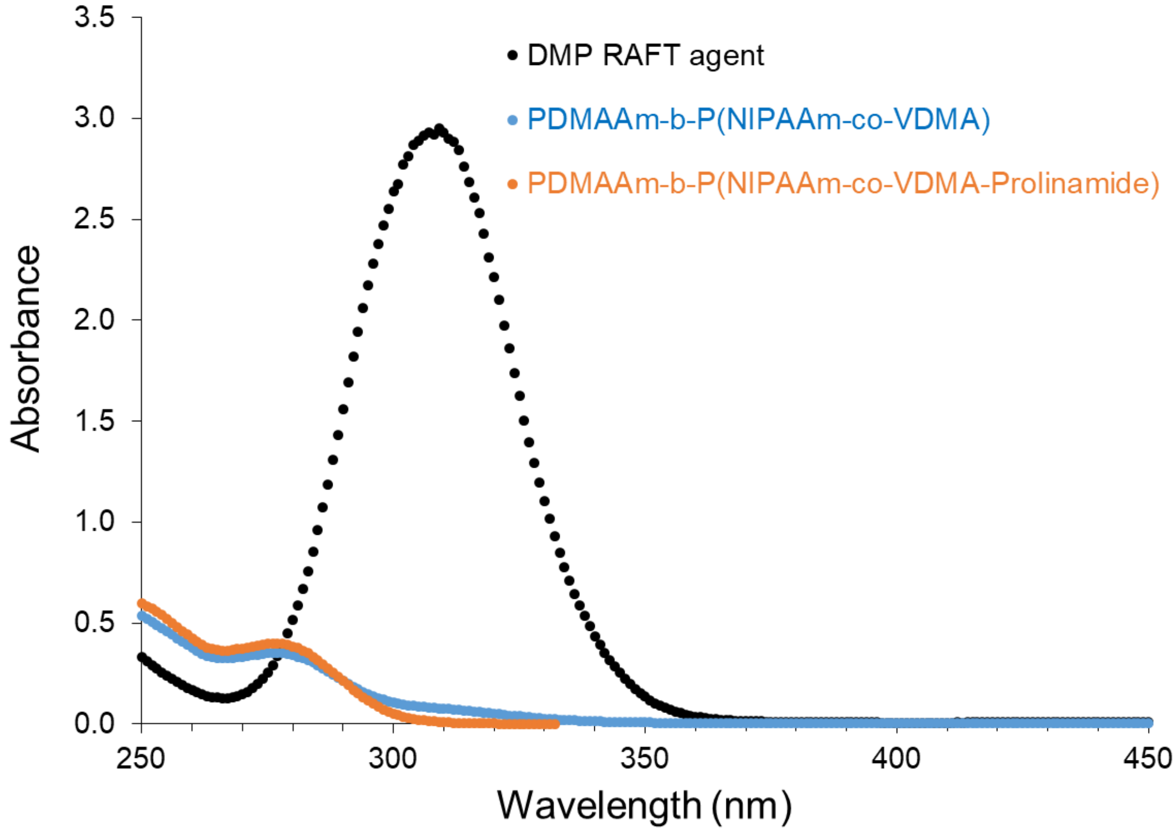

FIGURE S5: UV/VIS spectra of the DMP RAFT agent and the PDMAAm-b-P(NIPAAm-co-VDMA) block copolymer before and after attachment of the prolineamide organoctatalyst (solvent: methanol)
